# “Even if You Know Everything You Can Forget”: Health Worker Perceptions of Mobile Phone Text-Messaging to Improve Malaria Case-Management in Kenya

**DOI:** 10.1371/journal.pone.0038636

**Published:** 2012-06-13

**Authors:** Caroline O. H. Jones, Beatrice Wasunna, Raymond Sudoi, Sophie Githinji, Robert W. Snow, Dejan Zurovac

**Affiliations:** 1 Health Systems and Social Science Research, KEMRI-Wellcome Trust Research Programme, Kilifi, Kenya; 2 Nuffield Department of Clinical Medicine, Centre for Tropical Medicine, University of Oxford, Oxford, United Kingdom; 3 Department of Public Health and Primary Care, University of Oxford, Oxford, United Kingdom; 4 Malaria Public Health and Epidemiology Group, KEMRI-Wellcome Trust Research Programme, Nairobi, Kenya; 5 Center for Global Health and Development, Boston University, Boston, Massachusetts, United States of America; Johns Hopkins University, United States of America

## Abstract

This paper presents the results of a qualitative study to investigate the perceptions and experiences of health workers involved in a a cluster-randomized controlled trial of a novel intervention to improve health worker malaria case-management in 107 government health facilities in Kenya. The intervention involved sending text-messages about paediatric outpatient malaria case-management accompanied by “motivating” quotes to health workers’ mobile phones. Ten malaria messages were developed reflecting recommendations from the Kenyan national guidelines. Two messages were delivered per day for 5 working days and the process was repeated for 26 weeks (May to October 2009). The accompanying quotes were unique to each message. The intervention was delivered to 119 health workers and there were significant improvements in correct artemether-lumefantrine (AL) management both immediately after the intervention (November 2009) and 6 months later (May 2010). In-depth interviews with 24 health workers were undertaken to investigate the possible drivers of this change. The results suggest high acceptance of all components of the intervention, with the active delivery of information in an on the job setting, the ready availability of new and stored text messages and the perception of being kept ‘up to date’ as important factors influencing practice. Applying the construct of stages of change we infer that in this intervention the SMS messages were operating primarily at the action and maintenance stages of behaviour change achieving their effect by creating an enabling environment and providing a prompt to action for the implementation of case management practices that had already been accepted as the clinical norm by the health workers. Future trials testing the effectiveness of SMS reminders in creating an enabling environment for the establishment of new norms in clinical practice as well as in providing a prompt to action for the implementation of the new case-management guidelines are justified.

## Introduction

Between March 2009 and May 2010 a cluster-randomized controlled trial of a novel intervention to improve health worker malaria case-management practices was carried out in 107 government dispensaries and health centres in Kenya. At the completion of the trial a qualitative study was undertaken among health workers involved in the intervention to explore their perceptions and experiences of the process and to identify factors contributing to the observed outcome. This paper reports on the results of the qualitative study.

**Figure 1:**
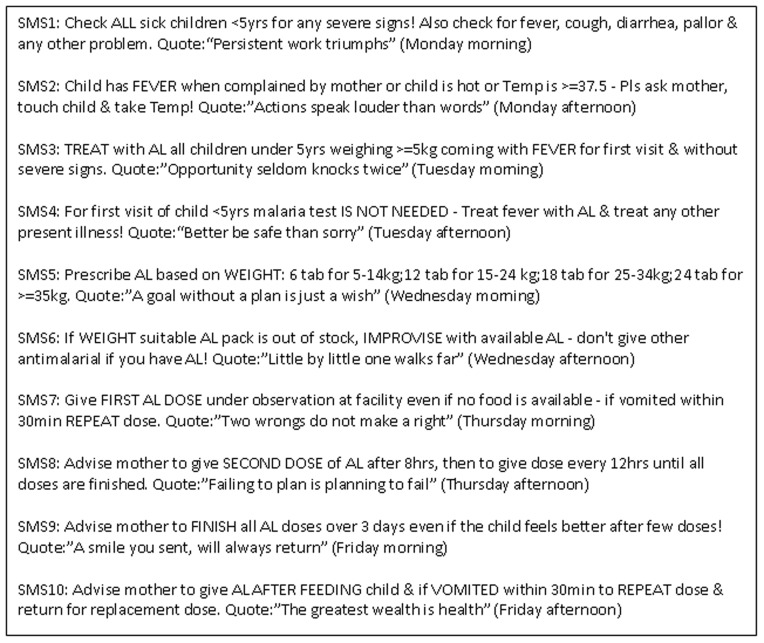
Content and schedule of text-messages. An example of the text-messages delivered during the first week of the intervention - while content and schedule of malaria component of the text-messages remained the same throughout the intervention period the quote component of the text-message was unique to each message sent to health workers.

The novel intervention consisted of sending text-messages about paediatric outpatient malaria case-management accompanied by “motivating” quotes to health workers’ personal mobile phones [1]. Ten malaria messages were developed (Figure 1). The accompanying quotes were unique to each message. The decisions on the content, number, length, order and the frequency of malaria messages considered recommendations in the national guidelines and training manuals, non-adherent practices observed in the previous studies, the maximum number of characters that can be sent in a single message, a logical order of an outpatient clinical process for febrile patients, and the desire to deliver all malaria messages reflecting a clinical process within a reasonably short period. These considerations resulted in sending two messages per day (9 am and 2 pm) for five working days (Monday to Friday) resulting in a total 10 different malaria messages weekly. Finally, the duration of 26 weeks of exposure was determined because changing clinical practices was considered a difficult task and a substantial repetition was felt necessary to eventually improve the practices.

The trial was undertaken in western and coastal areas of Kenya where artemether-lumefantrine (AL), the first line treatment for uncomplicated malaria, was introduced in 2006. Between 2006 and 2009 the AL implementation process in these areas included several rounds of case-management training for health workers including dissemination of new guidelines and wall charts. The intervention was delivered to 119 health workers at 54 health facilities. To assess the intervention three health facility surveys, with a cluster defined at health facility level, were performed: one before the intervention began, one at 6 months just after the intervention, and one 6 months after the end of the intervention. An intention-to-treat analysis showed a 23.7% improvement in correct artemether-lumefantrine (AL) management immediately after the intervention (November 2009) and sustained effect of 24.5% 6 months later (May 2010). The major improvements were achieved in the performance of the following four dispensing and advisory tasks that were rarely performed before the intervention: administration of the first AL dose at the health facility, advice to give the second AL dose after 8 hours, advice to give AL after a meal, and advice on what to do in case of vomiting. The performance of the six remaining tasks that were performed for the majority of children prior to the intervention had smaller effect sizes that were rarely statistically significant [1]. In comparison with many other interventions aimed at improving health worker case management practices in developing countries, these effect sizes are large and their maintenance post-intervention are noteworthy [2]. To inform the development of similar interventions in the future it is important to understand not only why the effect size is so large (why there was so much change in behaviour) and why these effects were so successfully maintained, but also how the intervention worked. That is, what were the features of the intervention that help to initiate and sustain the observed changes in behaviour?

## Methods

Twenty four health workers participated in in-depth interviews. They were purposively selected to include equal numbers of health workers from western and coastal study areas. Nearly all interviewed participants were nurses (21/24), two were community health workers and one was a clinical officer. Of 24 health workers 18 attended in-service training on AL management. The characteristics of interviewed health workers were similar to the universe of health workers exposed to the intervention during the main trial where nurses, community health workers and clinical officers respectively represented 80%, 13% and 7% of health workers while 62% were trained on AL management. Interviews were conducted in English between 16^th^ November and 2^nd^ December 2010 by one of the authors (BW). Each interview lasted between one hour and one hour 45 minutes and with the consent of the participants the interviews were recorded using a digital recorder. Written informed consent was obtained from all participants and the study protocol was approved by the University of Oxford Ethics Committee (OXTREC No. 3808) and the Kenya Medical Research Institute (SCC No. 1329).

A framework approach was adopted for both data collection and analysis where by the objectives of the data collection were pre-determined and structured topic guides were used in the interviews [3]. However, while a structured topic guide was employed to guide the interview process all responses were open-ended and the interviews were flexible allowing pursuit of issues raised by the participants that were not in the original topic guides. The interviews were transcribed and the transcripts entered into Nvivo 9 for data management and analysis [4]. Following familiarization with the data a thematic framework for coding was developed that reflected both questions raised by the project objectives as well as the issues raised by the participants. The interviews were coded using the framework and emerging themes were identified, cross referenced and examined to describe and interpret the impact of the SMS texting intervention.

## Results

### Perceptions of the Intervention

The 24 health workers interviewed for the study all showed an enthusiastic acceptance of the SMS text messaging intervention. It was perceived to be innovative, relevant and useful in clinical practice. The features of the intervention that were most frequently mentioned as positive attributes were: the conciseness of the messages, their accessibility and the fact that they contained up to date and pertinent information.

*Yeah it was very interesting and innovative; since by the time the messages were coming out we were new with AL drugs. It was a very nice maybe I can call it was a nice tool for improving my AL dispensing.* 5–56.

*You see that message is very short you read it within seconds and it had weight, it is helping you in your daily practice.* 6–09.

#### Frequency, Length and Timing of Messages

All of the participants said that the messages were an appropriate length (containing sufficient information delivered clearly & concisely) and the majority (18/24) were happy with the frequency and time of day of the messaging (twice a day, mornings and afternoon five days a week).

*It was clear because it was coming to remind me twice a day if in the morning it has reminded me and if I forgot within the day because of being busy and then in the afternoon still it reminds me don’t forget.* 6–19.

*The timing was very adequate in fact we were very happy, I was receiving a message very early in the morning I think it was nine and at least at four I received the other one. It was ok and it used to make me remember.* 6–11.

However, a few (6/24) were concerned that twice a day was too much and it could become ‘boring’. One of these six participants suggested that it wasn’t the number of times that the message was received that was the problem, rather that is was the same messages repeated too many times.

*Twice a day was too much I prefer in the mornings (only).* 5–52.

Another suggested that receiving them two or three times a week would be sufficient:

*The timing twice a day was too much…….Because like us we were aware of things but in the interior we have got a lot to do sometimes my message could come for lets say two days then I get tired of reading them. …. Otherwise they were very helpful a lot they were too many and I was hoping if I could get at least twice or thrice a week that one would be better.* 5–08.

While a third suggested that once a day (in the morning when work was starting) and early in the week were the most relevant times for receiving SMS messages:

*Ok sometimes it was too I felt it was too busy especially the ones which came in the evening because in the evening you expect now I am off from my place of work I should be receiving messages maybe from other people telling me where are you going are you at home. So I felt they would be more convenient if we were to receive them first thing in the morning while I am working morning hours when they know that I am working and I am a bit busy………Monday s and Tuesdays so those are the real days of hard work so those are the days when I need to be reminded because there is a long queue I cannot even go to the books to check.* 5–52.

#### Duration of Intervention

None of the participants complained about the length of time (26 weeks) over which the intervention was delivered and all expressed enthusiasm for a continuation of the SMS text message intervention. When specifically asked if they would like the SMS text messaging to continue all of the participants said that they would, but two participants qualified their answers by saying only if the SMS texts contained new information.

[Would you like to continue receiving the text messages?] *Yes*. [Would you prefer the same ten?] *No.*

[Why?] *Because already I know them*. 6–35.

In response to questions about possible new content the majority of participants mentioned that they would appreciate messages relating to malaria in pregnancy and severe malaria and when asked about other diseases the most commonly mentioned were pneumonia, ARIs, HIV and diahorrea. A few of the participants also mentioned non-communicable diseases such as diabetes and arthritis.

*Even on managing pregnant mothers; malaria in pregnancy if at least the messages on how to deal with them can be familiar on how to deal with the mothers who have come and malaria is positive.* 6–11.

*Ok there is HIV/AIDS also respiratory tract infections especially for children, diahorrea diseases I think those ones are enough.* 5–08.

Interestingly, while the majority of the participants had said that the messages they had received during the intervention had been reminders rather than new knowledge, when talking about additional messages or the continuation of messages the focus appeared to be on ‘updates’ and ‘updated’ knowledge, rather than reminders to practice.

*So long as if they have not changed it is ok you know they act as a reminder on out daily practices but if there is anything new, it is also ok to receive them; those messages.* 6–40.

*You know in my work I can say update is very vital, if you get malaria guideline/message and updates that will make a technician or a health worker to improve any management where there is malaria or other conditions*. 6–33.

*Like HIV/AIDS management there are so many things which are coming up every day so if there are new things that health workers need to know then it is good for us to get those messages.* 6–28.

#### Proverbs

The participants were asked about the inclusion of a proverb with the malaria guideline text messages. They were asked what role the inclusion of a new proverb with each messages had played in their willingness to read the text messages as well as on their overall perception of the intervention. All of the participants greatly appreciated the proverbs and there was some evidence that the new proverb each day did encourage people to read the next message, although none admitted to reading the texts for the proverbs alone. The most frequent response to the question ‘what made it interesting to want to read the messages’ was both the malaria content and the proverb, that is, a combination of the two.

*I think it was both because apart from the message these other sayings or proverbs you see they were just giving me, it’s like to be told that you will be told something tomorrow so you are anxious to wait and see what you will be told.* 5–42.

*Me I would say both because you see after reading the malaria content the quote also was giving me another message either to encourage me or it would make me laugh you see because some of which can make you laugh. They were interesting so they made me all the time I was thinking if I get a message I will get another quote, a different one.* 5–57.

Many of the participants did, however, suggest that the ‘entertainment’ of receiving the proverbs helped to ensure that the malaria messages were read.

*You know as I say food without salt is not tasty so I think they were trying to make us enjoy the message.* 5–21.

*Yeah there was some entertainment part of it some good sayings, I can say malaria content that message about malaria it had a lot of weight but the entertainment part also used to make you learn some new some sayings that you used to know to learn when you were in high school somewhere but you have forgotten……* 6–41.

While others linked the moral in the proverb to the content of the malaria message and perceived the proverb to be a way of reinforcing the clinical message.

*That the proverb was helping me to start thinking this proverb how is this proverb connected with this message and through that you even become concerned more.* 6–11.

*Through reading this message you see this message was the messages we very well understood then at the bottom you have these proverbs. From the message at least you have something that you will do then this proverb will add weight on the message. Ok the two were ok they could make me one could make me understand the other.* 6–35.

### Mechanisms of Action

In addition to examining the participants’ perceptions of the intervention and their experiences of receiving SMS text messages the interview data were also explored to identify any possible factors which may have contributed to changes in clinical practice that were recorded as an outcome of the trial. That is, the text were analysed with a view to identifying any possible ‘drivers of change’ which might help explain the quantified changes in practice that were observed as a results of the intervention.

### Messages as Reminders

A dominant theme emerging from the data was that, for the majority of the participants, the messages were an active reminder and a prompt to action to implement in their routine clinical practice the theoretical knowledge that they already have.

*Let me tell you the messages they were making me to do better because they were reminding me and when I came here before I had no knowledge on AL. I was taken to a seminar I was given knowledge and I forgot, when the message came they were reminding me.* 6–19.

The principal reason that all of the participants mentioned for needing a reminder was that, due to a heavy work load, they were often under pressure in their daily practice and this pressure had an impact on their delivery of clinical care. The messages were a reminder of the importance of implementing the practices referred to in the texts.

*The message actually it was something we knew before because it is even written in those packs of AL but for clinical setting sometimes you know somebody can forget when you are overworked, so it was quite relevant and as I said before trying to remind you.* 6–41.

*It was relevant you know sometimes you have work load and maybe you are alone in the facility so sometimes you might maybe seeing a patient without following the proper guideline so if you have just read the message it reminds you.* 5–26.

*Yeah sometimes we tend to be very busy and when the flow comes in you just treat without checking and if the child is very sick what you say you get the message then again you get another message then you tend to remember then you keep on practicing.* 5–08.

A few of the participants, primarily those who worked alone or in small dispensaries, also mentioned that it was not only pressures of a heavy workload that made them ‘forget’ best clinical practice but that factors external to the workplace, such as problems at home, might also impact on the quality of care they delivered. The messages acted as a daily reminder of the clinical work that needed to be undertaken.

*Yes, because we have social problems when you reach the facility you get a message you have to take at least remind yourself something has to be done you have been given information so you have to remove all those things you have on you to consider.* 6–33.

*It was a reminder to me I took it as a reminder because nobody is 100% perfect sometimes you might be getting stressed from elsewhere but when you get this message you remember what you are supposed to do to manage this child we are not 100% perfect.* 5–21.

#### Implementation of knowledge in practice

The participants all expressed the view that seminars or training course were important in terms of the acquisition of new knowledge and for motivation. However, it was clear that many of the participants believed that the type of training they received did not provide them with an adequate understanding of the importance of the new knowledge, or of the positive outcomes that a change in practice could bring. Once back in the clinic the pressures of day to day practice meant that not all the new knowledge was necessarily adopted as practice and became ‘forgotten’.

*Ok the important thing here I told you earlier that ok you kept on reminding us, kept on reminding us and we are seeing patients on daily basis and these messages are coming and we are picking them as we are seeing the patients. So it was very beneficial to us because immediately you leave from a seminar you don’t pick each and everything but as you practice as you continue practicing is now when you continue getting to know all these. So it acted as a booster.* 5–34.

*They are beneficial because as I told you most of these things we are actually aware of them through the seminars, trainings, the national guidelines but maybe the clinical settings when you reach here sometimes you do forget…. you even forget about teaching the client* ***……*** *so they are beneficial to me and the client in the side of me passing the information again to the client.* 6–41.

*…what the message was it is what we were taught in class when we went somewhere for a seminar about AL so it was working as the same way I was speaking from behind it was like a reminder if I had forgotten the kilos the message has brought the kilos to me, near to me at my desk it is just scrolling the phone then I get everything from there I continue prescribing according to the way it is needed to be done.* 6–19.

#### Clinical importance of a practice because of inclusion in the SMS messages

The qualitative data in this study suggest that it is those activities that are perceived to be less clinically important (e.g. giving advice about how to take the drugs) and/or those in which the clinician has less control over the outcome (e.g. make sure you finish the course of the drug) are those that are most likely to be ‘forgotten’ (or overlooked) if the workload is heavy. The data also suggest that putting the information into one of the messages emphasized its clinical importance which in turn encouraged its implementation in daily practice.

*Actually yes because at first before the messages after giving the start dose let them go home so we were less interested if weather they were going to take it or not but after these messages it sounded that it was very important for them to complete the doses so that to make the course complete.* 5–08.

*Lakini (but) when you are getting a message all the times it means you should be very very careful on that particular management.* 5–26.

This appears to have been particularly true for those messages which referred to counseling tasks. The quantitative data showed that of the four the biggest improvements in health worker practice: giving the first dose at the health facility; counseling to give the second dose after 8 hours; counseling to give the drug after a meal; and counseling about what to do if vomiting occurs, three were achieved in counseling tasks. This is reflected in the data from the interviews where the participants said that the messages relating to the counseling tasks had been the most useful and had resulted in the most noticeable change in their clinical practice. In particular, when the participants were asked to choose the most useful messages and those that had the most impact on their clinical practice, the most frequently mentioned (by 18/24 of the participants) was message 10 which covers two of four most improved practices, (advice mother to give AL after feeding child and if vomited within 30 minutes to repeat dose and return for a replacement dose). In addition, the one non-counseling task in which there was significant improvement was the dispensing of the first dose at the facility. This message (give first does under observation at facility even if no food is available – if vomited within 30 minutes repeat dose) was the second most frequently mentioned (17/24) when the participants were asked about the most useful message. By contrast, messages one to four which were commonly performed prior to the intervention (those dealing with diagnosis and prescribing) were rarely mentioned as being among the most useful or as having had any impact on clinical practice.

#### Creation of an enabling environment

There is some evidence in the data to suggest that for some of the participants the messages are seen as a form of support.

*Actually according to the messages to me one I was doing as the messages because to me as I said its like training they are trying to tell you do this because of this and also when you do something because it is the right thing to you, you feel contented because at least somebody is trying to remind you what you are supposed to do.* 5–21.

However, few participants mentioned the messages in terms of ‘support supervision’, rather that they were made to feel somewhat guilty for not employing proper practice and the receipt of the messages linked them in to a network.

*It was beneficial because at times you know you go for a workshop and you find I’ve gone to the workshop and whatever I have learnt is too much. But when this thing is done in a message you feel guilty like its there is somebody who is telling me do this you know because it is a message that comes when I am clerking a kid and I have just read it. It’s like you are feeling guilty like as if someone is telling me apart from the fever look at the kid it was making me do the right thing.* 5–52.

*Yeah at first ok I used to try my best to work as what it is supposed to be but the messages was like a supervisor to me so I felt bad if I do something which is not supposed to be done and there is someone who is reminding me every day it saves time and money also to remind you so I felt bad.* 5–08.

The dominant theme emerging from the texts was the importance of the messages as a reminder to practice, but there was also evidence of the importance of a sense of being ‘updated’. However, ‘updated’ seemed to be used in a variety of ways. Sometimes the term was used to emphasize the fact that the messages had helped them to ‘update’ their own practices (rather than provide new information).

*To me after getting the messages since I had already started practicing I was also happy because I knew at least I am being updated, that I am supposed to do this you know sometimes you might be having a lot of work and maybe you might decide to do some short cuts. But when you are being reminded all the time on remembering oh no I have just received the message so you will remember.* 5–26.

While for others the term was applied specifically to the receipt of new information.

*What made us to perform better than we used to before we started to receive the messages is actually nowadays things change very fast in health care, guidelines change about HIV,TB, Malaria and like that. Actually I was seeing it as an update; an update which I started to implement and because of implementing now it became a routine and actually by good luck we have enough supply of antimalarial drugs so at least now it made our work easy and now it became part of our clinical practice.* 6–41.

**Ok so would you think text-messages would be an easier way to update?** *It would be the easiest way because if they sit in Nairobi and they have changed because it is sms and the next morning I have started practicing and I am referring in my phone.* 5–52.

What is clear is that the messages provided motivation through giving the recipients the sense that they were linked into a network of information that connected them to both the national level policy and to other health workers who would be receiving the same messages and implementing the same practices.

### Relationship to Training, Guidelines and Other Interventions

None of the participants expressed the view that SMS text messages could be used to replace training and seminars as these were seen as essential to help in the interpretation and understanding of new guidelines, to give protected time away for learning (without the distractions of daily practice), to allow for an exchange of experiences with colleagues and to act as motivation (through staying in a nice hotel for a few days).

*The guidelines you know I can read a guideline and I don’t understand, I think if I go for a seminar I will understand better.* 6–40.

*In our facility it could be good but maybe in the hotels I can say it serves two purposes it acts as a motivation factor and also you learn and also its another place it’s a quiet place you can concentrate.* 5–42.

*Ok it is you know when we go for training it is not only that we learn from whatever that is coming new about malaria but we also learn from others maybe who are having challenges like me of consumers not finishing the drugs, of maybe children being left by with their young ones, maybe if we share experiences how can they also go back to my place and improve.* 5–52.

*I would like to go to these posh hotels with my colleagues in the district because if you come to…… you are going to see me alone but here we are going to meet with other officers from the district and now as we are learning we learn through one another, one is asking a question that is maybe beneficial to you and ask a question that is beneficial to me.*

On the other hand, the participants also pointed out some concerns with the current training system. Many felt that lecture based training alone didn’t give the participant the opportunity to practice what they had learn and so it was easy to forget in a real life setting.

*Practical is more because they say that somebody who just hears tends to forget earlier, quicker than the one who hears and sees and does for himself. So you will retain more when you hear, see and do.* 5–08.

*It should be a two way process there is this lecturing and then there is this practical of this kind because when you do it you don’t forget it…… So when you learn in the classroom you go out and the do it practically what you’ve learnt you keep it in mind and it is not easy to forget.* 5–42.

Some expressed the view that too often the workshops were poorly planned and rushed, trying to fit in too much information into too short a time, while others complained that some training kept people away from work for too long.

*Well you know sometimes there is those people who are facilitators might miss some of the important messages. Someone can run short of time and he or she tries to give a brief.* 6–54.

*The time was too short, time two seminars you know it is a marathon. It is a marathon a seminar that is supposed to take one week you are told it is two days so now you have to strain so at least they have to check the length of time it is inadequate.* 6–33.

The final concern with training was that it was often poorly timed. That is, workshop were held either too far in advance of the actual role out of a policy change (e.g. training on AL more than 12 months before AL became available), or too late (several month after a policy change so that health workers would use a new drug or test in the way they saw fit and by the time they went for training they would have already developed their own way of implementation, which might not be correct).

**Figure 2:**
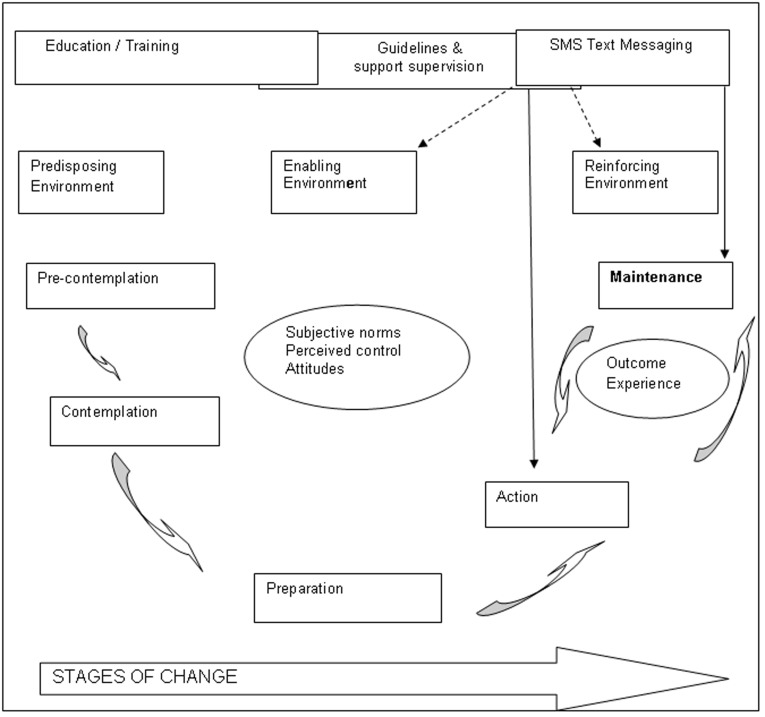
Conceptual framework describing the mechanism of action for SMS messages to change health worker behaviour. (Based on Prochaska & Di Celemente’s 1983 stages of change model).

Written guidelines are frequently produced and distributed to act as reminders of current policies on best clinical practice. However, the participants in this study perceived that guidelines were less effective in providing a reminder or reference than the SMS messaging primarily because they can be put in a drawer and forgotten. That is, guidelines are not so easily accessible firstly because the practitioner has to want to look at the guideline without a reminder to do so and secondly because even if the practitioner wants to refer to a guideline they have to physically locate them and then read through to find the appropriate advice, all of which takes time.

*And we are human beings and you can forget and you have no time to go through all these manuals and guidelines and whatever because of the work load that we have, so getting that kind of message helps you even helps you because you have no time to go to the guideline. Because now these messages we were getting and they are reminding you so they acted as reminders to us and they have assisted us very much.* 5–34.

*Normally we do not have time to go and refer that is the problem because of the integrated services we are rendering. You might be given a guideline you cannot even, you can even forget to go and refer but when you get a message directly it will wake you up; make you to wake up your mind that let me read this message regardless of referring to the, referring is a problem especially to health workers due to time and due to the work load we are having. But when you get a message it will assist you a lot but guidelines it will of course it is good you can refer but time limit.* 6–33.

In addition a person’s mobile phone is usually within easy reach and cannot be removed from the clinic (or lost) as is often the case with communally shared guidelines. Furthermore, retrieving text messages, if necessary, is quick and simple, and, as described in the previous section, information received via SMS text was perceived as being up to date whereas guidelines were always in danger of being rapidly outdated.

### Continuation of Effect: Creation of a Habit & Improved Outcomes

The repetition of the messages seems to act as a reminder to continue to implement the practices. The repetition of these clinical tasks then seems to become a ‘habit’ such that they are continued even after the messages have stopped being transmitted.

*It has been of help because you know when something is being repeated so many times it sticks into your head kwa hivyo (so) it’s not easy kubrush (to brush) off.* 5–26.

*It has been reminding us each and every day twice a day you should do this these things become now at our finger tips.* 6–26.

*You know when they keep on reminding me do this do this it becomes a day to day routine work.* 5–21.

*Actually I was seeing it as an update; an update which I started to implement and because of implementing now it became a routine.* 6–41.

The data also suggest that the health workers perceived that their changes in practices had resulted in positive impacts for themselves and their patients and consequently they were happy to continue to implement these practices.

*That message it helped me to build a relationship with the mother. ……the mothers were very happy to get those messages and most of them have improved…. when giving messages to mothers or clients you receive many more clients because they say you if you go to that daktari (doctor) he can give you a good message, he can observe the kid for the first time so that influences other clients to come in.* 6–33.

## Discussion

The results from the RCT demonstrated that SMS text messaging had a significant positive impact on health worker case management of malaria in febrile children [1] and this investigation of their perceptions of the intervention found that the SMS text messaging was enthusiastically received by the participants. The malaria content was perceived to be a very useful active reminder of ‘best practice’ and the new proverb with each SMS encouraged continued reading. All of the participants supported the fundamental idea of using SMS text messages to improve case-management practices but a few were concerned about the number of messages they received per week. On the other hand, they all reported that they would be happy to continue receiving text messages relating to the management of malaria and other common diseases. Acceptance by health workers of the SMS text messaging intervention appears to be high when compared to the results of a recent review of studies on the use of SMS text messaging for behaviour change among patients in clinical care and for clients in preventive health behaviours interventions. The review found participant retention ranging from 43% to 100% and great variability in acceptance and compliance with the SMS programmes [5]. However, the acceptability of the intervention to health workers in the current study is in line with the findings of two recent projects investigating the impact of interventions based on the use of mobile phone technology to improve the performance of community health workers (CHWs). The first project involved introducing multimedia (sound and pictures as well as text) to a mobile phone system to support CHW practice in India [6]. The authors report that, as was found in the current study, the key features of the system that made it appealing and useful were its convenience and portability (easier to access and less bulky than conventional job aids), its novelty and the perception of the messages having come from an ‘expert’ [6]. The second study, undertaken in Tanzania, found a significant improvement in the target behaviour (increase in timely home visits by CHWs) and considerable enthusiasm for the intervention among the CHWs and their supervisors [7]. The CHWs reported that they would have been happy to received more frequent messages (an average of 18.7 texts was sent to each CHW over a 40 day period) and that their preferred time of day for receipt of the messages was in the morning to help them plan their day [7]. However, neither the Indian nor the Tanzanian study was able to address the issue of the number of messages that need to be sent and over what time period in order to achieve the desired outcomes. This lack of evidence on the most effective timing, frequency and duration of messaging required to facilitate behaviour change was also highlighted by Fjeldsoe and colleagues in their review [5]. That is, among the SMS text messaging studies there are significant variations in terms of the method of intervention introduction, frequency of message delivery, duration of messaging, and SMS interaction with participants. To ensure the future development of effective interventions, more research is required to evaluate the effects of specific SMS characteristics (e.g. number of messages, time of day of delivery, number of messages per day/week, duration of repetition of particular messages etc) on health worker performance outcomes.

Evidence from many studies in sub-Saharan Africa has demonstrated that in-service training alone is insufficient to ensure changes in clinical practice and any improvements after training disappear quickly if no follow-up support is provided [8], [9], [10], [11], [12], [13] and yet in the public sector in sub Saharan Africa training with the distribution of guidelines is still often the only intervention provided to change clinician behavior [14], [15], [16]. The data collected in this study suggest that the participants themselves are well aware of the limitations of relying on training to change their clinical practice and they support the findings from a Tanzanian study on clinicians’ behaviour which found that one of the reasons training might be particularly ineffective in resource poor settings is that the motivation to attend trainings often lies in the extra payments received rather than the goal of attaining new knowledge [17]. In addition, selection for training was perceived to be biased and not based on need or qualifications [17]. However, the lack of impact of in-service training on health worker practices is not restricted to clinicians in Africa. A mapping review of the literature on interventions to improve clinician behaviour undertaken by Robertson and Jochelson for the National Institute for Clinical Excellence (NICE) in the UK found that there was no evidence that educational material alone had any impact on clinician behaviour [18]. On the other hand, the NICE review did find that appropriately designed and implemented education interventions may be a prerequisite for change to occur. The authors reported that workshops and small training sessions had been found to be more effective than large-scale didactic meetings but there was little evidence on whether the short-term effects of such meetings could be sustained in the longer term. A study by Rowe and colleagues on a multifaceted intervention implemented in Benin to improve health workers adherence to the guidelines for the integrated management of childhood illness also found that training was useful but additional post-training supports resulted in additional and more sustained improvements [12]. One form of support that, in the developed country context, has been shown to be generally effective in changing clinician behaviour are reminders to undertake or avoid a certain action [18]. While the NICE review found no evidence to suggest that reminders made an impact on diagnostic practices, they were found to be more effective in changing prescribing and general clinical management practices. These reminders had most impact when they were given at the point of decision making. The SMS text messages in this study were delivered at, or close to, the point of decision making, and the majority of the participants referred to them as a reminder to practice. Furthermore, the behaviours on which there was most effect were those relating to prescribing and general clinical management.

Environmental support has also been found to be central to encouraging adoption and maintaining adherence to guidelines among clinicians [6], [19]. The data from this study suggests that SMS text messages helped integrate guidelines into the process of care, not only through supplying active reminders to the participants, but also through enhancing the perception that their clinical practice was being undertaken in a supportive environment. Several studies have shown that, where available, health workers rely to a large extent on interactions with colleagues, opinion leaders and patients to inform their practices [17], [20]. In the context of the delivery of health care in rural Kenya many health workers are isolated from a broader clinical community and interactions with peers, opinion leaders and other possible sources of information on best practice are severely curtailed by logistical and economic constraints. This sense of isolation is one of the reasons that health workers are keen to attend trainings as it gives them a chance to interact with their peers. While text messaging does not provide active interaction it does appear to be filling some of this communication gap by linking the recipients into a network of information on best practice that is perceived to be authoritative, up to date, relevant to their day to day practice (validating and supporting their actions).

The participants’ belief in the validity, authority and clinical importance of the messages as well as on their willingness to read them may have been influenced by the mode of intervention initiation. That is, the messages were seen to be the best and most up to date evidence from a trusted source, the Kenyan Medical Research Institute (KEMRI) in collaboration with the Ministry of Health (MOH). Guidelines have been found to be more acceptable to clinicians if they are endorsed by a respected peer and senior management and it could be that, in this intervention, receiving the reminders from ‘KEMRI’ provided the endorsement which facilitated their adoption into practice [21], [22]. The findings in this study do not provide evidence on the extent to which the perceived involvement of KEMRI influenced the participants’ willingness to take note of and implement the practices mentioned in the SMS text messages. An evaluation the intervention as it was rolled out under routine conditions would be required to allow for an assessment of the influence of this effect on the observed outcome.

This SMS study is one of the many studies aimed at changing health worker behaviour and improving adherence to guidelines that have been implemented in sub-Saharan Africa in recent years. The outcomes of these efforts have been documented and widely disseminated such that we are starting to get a view of what works and, just as importantly, what doesn’t [11], [12], [13]. However, there is little information or discussion in this literature on the drivers of change, the possible mechanisms through which interventions might be acting in order to bring about any observed improvements. An approach to understanding the process of behaviour change that has been used in the development and implementation of health promotion initiatives globally and in promoting the use of evidence in clinical practice in developed country settings is the transtheoretical (stages of change) model [19], [23]. The model was first proposed by Prochaska and Di Clemente [24] and while there is little evidence to demonstrate that its use has been particularly effective in increasing the impact of behaviour change interventions [25], [26] it is a useful tool in helping to identify which health worker behaviours might be most amenable to the influence of particular types of intervention such as the SMS text messages. The framework describes change as a process with participants moving from pre-contemplation, through contemplation, to decision, then active change and maintenance. Using the framework the participants location in the change process for any given behaviour can be identified. Interventions and strategies appropriate for moving the participants from the current stage to the next can then be developed and implemented [23]. For example, to move from pre-contemplation participants would need information and this might be best delivered through training and seminars given by respected peers. To move from contemplation to action participants need to be convinced that they have sufficient control to implement the change, that the change is acceptable to peers and seniors and that it will have positive impact with little risk. This shift will be facilitated by practice based training but also requires interactions with and support from colleagues and seniors in the establishment of new behavioural norms. Reminders may be needed to maintain action and action is reinforced by positive outcomes.

Applying the framework to this SMS intervention the messages can be seen to have been operating primarily at the action and maintenance stages of behaviour change (Figure 2). In addition, the messages help to create an enabling and reinforcing environment, facilitating the uptake of normative behaviours through improved networking and communication. That is, the health workers had largely moved beyond the stage of needing to be convinced that the prescribing, dispensing and counseling messages should be followed (the recipients had already contemplated and prepared for change through training) and had accepted that these practices were normative behaviours. Thereafter, what the SMS text messages did was to encourage the practice of these behaviours (a prompt to action) as well as providing support to practice (reinforcement for action). The SMS messages were acting as both a reminder for action and support to maintaining behaviour. The two most prominent factors that are likely to have contributed to continuing practice once the messaging had stopped are likely to have been the development of a ‘habit of practice’ which was reinforced by the positive outcomes experienced through the adoption of the new behaviours. What is unclear, from both the results of this study & from the broader literature on the use of SMS text messaging in health behaviour change, is the frequency and duration of messaging that is required to create a new ‘habit’ in clinical practice [5]. The data do show that the delivery of two messages a day on 5 days in a week for 26 weeks is sufficient to create new habits and facilitate a sustained change in practice. Using this information, one SMS text messaging strategy worth testing is ‘pulse-messaging’. That is, focusing on daily messages related to a specific disease, type of practice, or other public health topic, delivered at the start of the working day on the five working days of each week for a period of 6–8 weeks, followed by an interruption of 6–8 weeks, followed by a fresh series of messages on a different disease or topic.

While the SMS text messaging has proven to be very successful in improving health worker adherence to current malaria treatment guidelines, in many countries in sub-Saharan Africa those guidelines are changing (or have already changed) from presumptive treatment to parasitologically confirmed treatment. The new guidelines contain two major differences compared to presumptive recommendations. First, febrile patients should be tested for malaria and second, only test positive patients should be treated for malaria [27]. While there have been a few exceptions [28], [29] initial routine implementation experiences have generally shown low levels of testing [16], [30], [31], [32] and high levels of non-adherence to test negative results [30], [33], [34], [35]. With respect to the former, we hypothesize that text-message reminders may be immediately effective to prompt action and increase testing rates because the use of testing is not conflicting with the traditional parasitological concept of malaria; the health workers knowledge of this concept is widespread, and environment of respected peers and seniors but also patients is likely to be supportive of such practices [17], [36]. Moreover, in developed countries reminders had been shown to increase adherence to test ordering guidelines [18]. Our hypothesis, with respect to adherence to test-negative results, is that health workers are still at the contemplation phase. That is, they are not yet convinced that malaria really is in decline and, despite some training in the use of RDTs, in absence of a strong enabling environment to support their decisions and the lack of alternative treatments for test negative patients, they are not convinced that withholding malaria treatment is indeed safe and presents a normative (culturally acceptable/ideal standard) behavior [17], [36], [37]. Text message reminders in this area of clinical practice might help to strengthen the enabling environment by creating a real or perceived links among recipients and by providing supportive information from a respected source. However, we hypothesize that the full potential of the text message reminders in this area of practice, is likely to be achieved only if additional interventions are put in place to minimize these concerns in order to facilitate the transition from contemplation into action. In the real world of weak health systems where key enabling interventions such as supportive supervision and quality assurance processes are rarely adequately implemented, only rigorously evaluated studies in a variety of operational settings may prove or disapprove our hypotheses and reveal the effectiveness of text-message reminders in improving this common deficiency in the practice.

## Conclusion

The data from this study suggest that SMS text messaging as an intervention is an acceptable and effective way of providing front line health workers with active reminders of best practice for malaria case management. However, more research is required to establish how frequently messages need to be repeated and over what time period the same messages need to be delivered in order to establish new habits of practice. Based on the results we would suggest that key factors likely to influence the acceptability and uptake of any future SMS messaging interventions include the perception that the messages are developed by a trusted authority, that they are relevant to clinical practice and that they are delivered at, or close to, the point of care. Furthermore, we hypothesize that it is unlikely that, without additional supportive interventions, SMS text messaging will be as successful in changing case management practices that require a shift in clinical norms (e.g. not treating test negative patients for malaria) as they have been in prompting the implementation of practices which are already widely accepted by front line health workers as being best clinical practice. Future trials testing the effectiveness of SMS reminders in creating an enabling environment for the establishment of new norms in clinical practice as well as in providing a prompt to action for the implementation of the new case-management guidelines, are justified. In addition, as the use of mobile technology to improve the quality of health care grows, on-going operational research will be required to refine, tailor and monitor how people respond to repeated messaging. Of particular concern is to understand what happens when such technologies and innovative interventions are no longer novel and exciting.
